# Basic life support training for lay students from a public university

**DOI:** 10.1186/cc14672

**Published:** 2015-09-28

**Authors:** Silmara Meneguin, Debora G de Santana, Larissa M Noda

**Affiliations:** 1 Medical School of Botucatu, UNESP, Distrito de Rubião Jr S/N, Botucatu, São Paulo, Brazil

## Introduction

Emergency care situations come up in people's lives unexpectedly, demanding rapid, objective and effective action. People are not always properly trained to deliver this care though.

## Objective

To attend to this shortage, this study compared the psychomotor skills and theoretical knowledge of lay students in the cardiopulmonary resuscitation (CPR) technique before and after training.

## Methods

Concurrent multiple cohort study, based on a sample of 424 students from a Brazilian public university who participated in on-campus training between July 2012 and December 2014. To assess the theoretical knowledge, a structured questionnaire was used and, to assess the psychomotor skills, a checklist with the steps of the CPR technique, in line with the 2010 guidelines of the American Heart Association. The participants were divided into four knowledge areas: biological, exact, agricultural and health.

## Results

These study results evidenced a statistically significant increase in correct answers on the theoretical knowledge and psychomotor skill assessment tools after the training in relation to the areas. Exact and agricultural sciences were the areas that most evolved in terms of the number of correct psychomotor skills. Agricultural and biological sciences were the areas that most evolved regarding theoretical knowledge about cardiopulmonary resuscitation. Before the training, the mean number of correct skills was an average 1.79 points higher for each additional year of age and the men's score was 6.6 points lower than the women's score. After the course, only the relation between age and number of correct skills continued significant and gained strength. For each additional year of age, the number of correct skills increased by an average 8.21 points. As regards the theoretical knowledge score on CPR, before the course, a significant relation existed between age and having taken the first aid course. The score increased by 0.22 points for each additional year of age and was 0.63 points higher among participants who had taken the course earlier. After the training, sex and having taken the course earlier remained significantly related with the theoretical knowledge score on CPR.

## Conclusion

These study results indicate that the participants have presented improvements in their performances. After the training, the increase in the number of correct answers on the psychomotor skill tool was directly proportional to the age. Concerning the theoretical knowledge on CPR after the course, age and having taken the first aid course contributed to increasing the number of correct answers.

